# Supplementary material for: Rapid turnover of life-cycle-related genes in the brown algae
Source: Genome Biol. 2019 Feb 14;20:35. doi: 10.1186/s13059-019-1630-6 (PMC6374913; doi:10.1186/s13059-019-1630-6)
Supplement: Supplementary file 3 — Protein alignments, corresponding Gblocks html file and CDS sequences for the lineage conserved gametophyte-biased genes. (ZIP 2109 kb) [file 13059_2019_1630_MOESM3_ESM.zip › Con_GA_alignments/Ec-22_000580.aa_ali.fasta-gb.htm]

Ec-22\_000580.aa.fasta.trans.aa\_ali.fasta


## Gblocks 0.91b Results

Processed file: **Ec-22\_000580.aa.fasta.trans.aa\_ali.fasta**  
Number of sequences: **2**  
Alignment assumed to be: **Protein**  
New number of positions: **313** (selected positions are underlined in blue)

```
                         10        20        30        40        50        60
                 =========+=========+=========+=========+=========+=========+
Ec-22_000580     ---MRRPG---WAFCVEALLLLLVLPRAAGGTAFFSRRRSLQVLTDGETVLNGVLNLFTE
MSTRG.26837.1    RVPPRDPNPCERAMARLLLTVLLLLGSLAVADAAPARR-SLQVLLAGDTVVNGVLNLHTS
                             ########################## #####################


                         70        80        90       100       110       120
                 =========+=========+=========+=========+=========+=========+
Ec-22_000580     EGVAACEGSCKDAFATAFGIDEVSVGCVCPISTSASDADRRRNLMGDNSKQPTSDSAEQN
MSTRG.26837.1    EGVDACESSCKPAFAEALGVAEGAVGCLCPISANSSATS--------SAEEEGSVRVRRN
                 ####################################                 #######


                        130       140       150       160       170       180
                 =========+=========+=========+=========+=========+=========+
Ec-22_000580     KGSSDRRSLRAQRRRWLAETDSSEPVAFSARVAGGLAGGAAALDLLSESGWSAVASAFGV
MSTRG.26837.1    LRHEQRHRNSVR---RLAELEAGEPVFFSARVEGGLDGGATAVENFSVSGWSIVASAFGV
                 ######          ############################################


                        190       200       210       220       230       240
                 =========+=========+=========+=========+=========+=========+
Ec-22_000580     DEDELEISTLTVTPPASTNSTETLEVITDYFDASDISIVSSLSTTEGTDWAGGGRGVALF
MSTRG.26837.1    QESEMDITTLTVTPPASSVANETLTVITDYFDASDIAILP----IHETDWAGGAKGAALF
                 ######################################         #############


                        250       260       270       280       290       300
                 =========+=========+=========+=========+=========+=========+
Ec-22_000580     LAMFVLLLMTFCCCGFLAGLLLWKAKKHGGGAVGGEKFFKSHLPERNSFRWGGQPTADGN
MSTRG.26837.1    LALLVLVVGTCCCGGLLALGLLWKVRKDAGGDS--KKFLFLERKSSVTEQSGGPAKNFER
                 ###############################                    #########


                        310       320       330       340       350       360
                 =========+=========+=========+=========+=========+=========+
Ec-22_000580     PRRSEPHSSEGASPNGPLLGLVDSQDSSNRSPSRENVPHEGDEAAGGVVDHVRATSASSA
MSTRG.26837.1    PERR----------------------SSRRQPAVDTNP--------------RRSHSSSD
                 ###                       ############              ########


                        370       380       390       400       410       420
                 =========+=========+=========+=========+=========+=========+
Ec-22_000580     AVAPESSSKSRGVSSGDLGKELESPSAAGRGGVTEQTAAAAAGAPAATAGGRDEEGAIPR
MSTRG.26837.1    VVSPSDSAFGLVRESG------AEPSPSGKGKDTEKLVAAPREGGSVARGG---------
                 ################        ################                    


                        430       440       450       460       470       480
                 =========+=========+=========+=========+=========+=========+
Ec-22_000580     NVSMEESVGTNPLYKTAAPYRRSTNESQSEQTPGRSRFSTFLGNATPSRLRRLAGRVAKS
MSTRG.26837.1    ----DEAAGGQ------------------------------------------GGEVSRR
                                                                       ######


                        490       500       510       520       530       540
                 =========+=========+=========+=========+=========+=========+
Ec-22_000580     PALSASSRRSKPSTASRHSRSGGSSTRSRRHRRQGSFPGAYSQRNSSELDHQRPPADPVY
MSTRG.26837.1    AVASS-----APDAAGHGGTVGGDVIMS---------PGA--------------------
                 ####       #################                                


                 
                 =========
Ec-22_000580     TFMIKSFDH
MSTRG.26837.1    -------RG
```

```
Parameters used
Minimum Number Of Sequences For A Conserved Position: 2
Minimum Number Of Sequences For A Flanking Position: 2
Maximum Number Of Contiguous Nonconserved Positions: 8
Minimum Length Of A Block: 10
Allowed Gap Positions: None
Use Similarity Matrices: Yes
```

```
Flank positions of the 11 selected block(s)
Flanks: [13  38]  [40  96]  [114  126]  [137  218]  [228  271]  [292  303]  [327  338]  [353  376]  [385  400]  [475  484]  [492  508]  

New number of positions in Ec-22_000580.aa.fasta.trans.aa_ali.fasta-gb:  313  (57% of the original 549 positions)
```
